# Frequency of urinary tract infection and antibiotic sensitivity of uropathogens in patients with diabetes

**DOI:** 10.12669/pjms.35.6.115

**Published:** 2019

**Authors:** Kaleem Ullah Zubair, Abdul Haleem Shah, Asher Fawwad, Rubina Sabir, Anum Butt

**Affiliations:** 1 Kaleem Ullah Zubair, M.Phil Scholar, Department of Biological Sciences, Gomal University, Dera Ismail Khan, Pakistan; 2 Abdul Haleem Shah, Dean, Department of Biological Sciences, Gomal University, Dera Ismail Khan, Pakistan; 3 Asher Fawwad, Ph.D. Chairman and Professor of Biochemistry, Department of Biochemistry, Baqai Medical University, Research Director, Baqai Institute of Diabetology and Endocrinology, Baqai Medical University, Karachi, Pakistan; 4 Rubina Sabir, M.Sc, Laboratory Manager, Department of Clinical and Research Laboratory, Baqai Institute of Diabetology and Endocrinology, Baqai Medical University, Karachi, Pakistan; 5 Anum Butt, M.Phil, Research Officer, Research Department, Baqai Institute of Diabetology and Endocrinology, Baqai Medical University, Karachi, Pakistan

**Keywords:** Antibiotics sensitivity, Diabetes Mellitus, Frequency of UTIs, Uropathogens

## Abstract

**Objective:**

To determine the frequency of urinary tract infections and antibiotic sensitivity among patients with diabetes.

**Methods:**

This observational study was carried out in Microbiology Department of Baqai Institute of Diabetology and Endocrinology (BIDE), Baqai Medical University from April 2015 to June 2016. All patients with diabetes having symptoms of UTI attending out patients department of BIDE were analyzed. All samples received in the laboratory were processed according to Clinical and Laboratory Standards Institute (CLSI) guidelines. Antimicrobial susceptibility pattern was determined by disc diffusion method.

**Results:**

A total number of 199 urine specimens, frequency of UTI were 24 (12.06%) in male and 175 (87.94%) in female. UTIs were highly found in (age group 51-60) 70 (35.18%). Escherichia coli was the most frequent pathogen (71%), followed by Klebsiellapneumoniae (7.48%), Proteus mirabilis (1.87%), Staphylococcus aureus (9.35%), Candida (5.61%) and Candidaalbicans were (2.80%). Majority of gram negative uropathogens were shown high sensitivity towards Imipenem and Piperacillin / Tazobactam followed by Nitrofurantion, Ceftriaxone, Levofloxacin, Ofloxacine, Ciprofloxacin, Norfloxacin, Cefixime, Nalidixic acid and Cephradine. Gram positive was most sensitive to Nitrofurantionand Vancomycin followed by Piperacillin / Tazobactam, Imipenem, Cephradine, Ceftriaxone, Norfloxacin and Cefixime.

**Conclusion:**

We observed the higher frequency of UTIs in female as compared to male participants due to poor hygiene. *E.coli* was the most frequent pathogen responsible for UTI in patients with diabetes, followed by Staphylococcus aureus.

## INTRODUCTION

Diabetes mellitus (DM) is now becomes a global health issue to health care professionals.^1^ According to International Diabetes Federation (IDF) 451 million (age 18-99 years) people was reported with diabetes worldwide 415 million people in 2017, which expected to increase to 693 million) in 2045.^2^ Approximately 7.5 million Pakistani people suffer from DM.^3^ Patients with diabetes are at high risk of urinary tract infections (UTI) and its complications especially in patients with Type-2 diabetes. A survey reported UTI to be the most common microbial infection worldwide.^4^ Globally, it was estimated about 150 million people each year affected from this infection. It is most common in women than in men^5^-^7^ and about 50-60% of women suffering from it at least one time during their lifetime.^5^ However, UTI pose a clinical problem elevates the risk of pyelonephritis, premature delivery, and fetal mortality among pregnant women, and is associated with impaired renal function and end-stage renal disease among pediatric patients.^8^,^9^

Similarly risk of UTI increases with age, poor metabolic control, various impairments in the immune system and incomplete bladder emptying due to autonomic neuropathy.^6^,^7^ The most contributed pathogen of UTIs is Escherichia coli in diabetic and non diabetic patients and others are Klebsiella pneumonia (K.pn), Staphylococcus saprophyticus, Proteus mirabilis, Enterococcus faecalis, Group-B Streptococcus, Pseudomonas aeruginosa, Candida spp, and Staphylococcus aureus.^4^ The microbes causing infection differ in their susceptibility towards antimicrobial drugs from place to place.^6^,^10^ The emergence of multi-drug-resistant (MDR) strains is escalating causing urinary tract infections increasing both in community and hospital settings.^6^,^7^,^10^,^11^ Health care professionals and physicians must acquired the knowledge regarding the frequency of different microorganisms and antibiotics susceptibility, so the proper antibiotics for treating the infection could prescribed. It is a big challenge in developing countries like Pakistan due to high incidence of infection, and irrational uses of antibiotics.^7^

As the continuous screening of trends and susceptibility pattern of predominant organisms against antimicrobials is essential therefore this study was designed to quantify the incidence of UTIs, pathogen involve and antibiotic susceptibility among patient with diabetes.

## METHODS

This observational study was conducted in Microbiology Department of Clinical & Research Laboratory of Baqai Institute of Diabetology and Endocrinology (BIDE), Baqai Medical University from April 2015 to June 2016. Ethical approval of this study was obtained from Institutional Review Board (IRB) of BIDE. UTI was generally asymptomatic in diabetics. Patients with known diabetes and clinically diagnosed UTI were included (through urine DR and culture sensivity report). Data regarding age, gender, duration of diabetes and HBA1c were obtained from hospital management system. Patient who take antibiotic was excluded. All proven diabetics with the level of HbA1c ≥7.0 gm/dl. Data were categorized into 7 age groups; Group-1 (20-30)yrs, Group-2 (31-40)yrs, Group-3 (41-50)yrs, Group-4 (51-60)yrs, Group-5 (61-70)yrs, Group-6 (71-80)yrs, Group-7 (81 and above)yrs.

Gram negative organism were identified using TSI (triple sugar iron), Citrate utilization, SIM (Sulphideindole motility media) and urea hydrolysis.^8^ Midstream urine (MSU) sample was collected^6^,^12^ and inoculated on three types of media: blood agar, MacConkey agar and CLED agar (Oxoid, Basingstoke, UK).^12^,^13^ All inoculated specimens were incubated at 37°C for 24–48 hours for visible growth. Organisms were identified by colony morphology, Gram stain and chemical tests like catalase, oxidase, coagulase TSI (triple sugar iron), Citrate utilization, SIM (Sulphide indole motility media) and urea hydrolysis. SDA (Sabourads Dextrose Agar) was used for the growth of Candida. Germ tube method used for the identification of C albicans.^11^,^14^

Antibiotic sensitivity test was done according to CLSI guidelines, on Mueller Hinton agar by Kirby-Bauer method.^11^,^13^ For antimicrobial susceptibility testing (AST) the discs used were Ciprofloxacin (5mg), Norfloxacin(10mg), Nalidixic acid (30μg), Nitrofurantion (300μg), Cephradine (30μg), Cefixime (5 μg), Ceftriaxone (30mg), Levofloxacine (5 μg), Oflaxacin (5 μg), Vancomycin (30μg), Imipenem(10μg) and Piperacillin / Tazobactam(110μg).

### Statistical Analysis

Analysis of data was done on Statistical Package for Social Sciences (SPSS), version 13.0. Data presented in the form of frequency and percentage.

## RESULTS

A total number of 199 urine specimens, 24 (12.06%) male and 175 (87.94%) were female diabetic patients who had clear signs of UTI in this study. The age of patients ranges between 20-93 years. Most of patients with diabetes with UTI were in Group-4 (age ranges 51-60) 70 (35.18%), followed by Group-5 (age ranges 61-70) 47 (23.62%) and Group-3 (age ranges 41-50). Female shown (87.94%) more UTI than male (Table-I).

**Table I:** Frequency of UTI according to gender and age.

Gender wise Distribution of Groups		

Gender	Frequency of UTI	Percentage of patients (%)
Female	175	87.94
Male	24	12.06

*Age wise Distribution of Groups*		

Group-1 (20-30)	19	9.55
Group-2 (31-40)	12	6.03
Group-3 (41-50)	36	18.09
Group-4 (51-60)	70	35.18
Group-5 (61-70)	47	23.62
Group-6 (71-80)	12	6.03
Group-7 (81 and above)	3	1.51

Out of 199 urine specimens 105 (52.76%) specimens showed growth of microorganisms (Bacteria and yeast). Total 107 isolates including 98 (91.59%) bacteria and 9 (8.41%) yeast were isolated. One hundred and three monomicrobial and 2 polymicrobial cultures obtained from 199 Urine specimens. Escherichia coli (*E.coli*) was the most common among Gram negative isolate 77 (71.96%) of total isolates followed by K.pn 8 (7.48%) and Proteus mirabilis (P.mir) 2 (1.87%). The most common Gram-positive isolate was Staphylococcus aureus (S.aur) 10 (9.35%), while frequency of Candida species was 6 (5.61%) and Candida albicans 3(2.80%) Entc ssp (1)0.93%. On the basis negative germ tube test Candida species identified.

*E. coli* showed higher sensitivity toward Imipenem and Piperacillin / Tazobactam (100%) followed by Nitrofurantoin (93.24%), Ceftriaxone (45.93%), Levofloxacin (41.33%), Ofloxacine (40%), Ciprofloxacin (38.96%), Norfloxacin (37.33%), Cefixime (37.33%), Nalidixic acid (28%) and Cephradine (20%) (Table-II).

**Table II:** Antibiogram of bacteria causing UTI in Diabetes patients.

Antimicrobial agents	Susceptibility	E. coli (%)	S.aur (%)	K.pn (%)	P mira (%)	Ent. Spp (%)
Imipenem (10 μg)	S	100.00	90.00	100.00	100.00	--
	R	0.00	10.00	0.00	0.00	--
Piperacillin / Tazobactam (10/100 μg)	S	100.00	90.00	100.00	100.00	--
	R	0.00	10.00	0.00	0.00	--
Nitrofurantion (300 μg)	S	93.24	100.00	62.50	50.00	100.00
	R	6.76	0.00	37.50	50.00	0.00
Ceftriaxone (30mg)	S	45.95	50.00	62.50	100.00	--
	R	54.05	50.00	37.50	0.00	--
Levofloxacine (5 μg)	S	41.33	30.00	75.00	50.00	0.00
	R	58.67	70.00	25.00	50.00	100.00
Oflaxacin (5 μg)	S	40.00	30.00	75.00	0.00	0.00
	R	60.00	70.00	25.00	100.00	100.00
Ciprofloxacin (5mg)	S	38.96	30.00	75.00	50.00	0.00
	R	61.04	70.00	25.00	50.00	100.00
Norfloxacin (10mg)	S	37.33	33.33	75.00	0.00	0.00
	R	62.67	66.67	25.00	100.00	100.00
Cefixime (5 μg)	S	37.33	10.00	50.00	50.00	0.00
	R	62.67	90.00	50.00	50.00	100.00
Nalidixic acid (30 μg)	S	28.00	22.22	62.50	0.00	0.00
	R	72.00	77.78	37.50	100.00	100.00
Cephradine (30 μg)	S	20.00	50.00	25.00	0.00	0.00
	R	80.00	50.00	75.00	100.00	100.00
Vancomycin (30 μg)	S	--	100.00	--	--	100.00
	R	--	0.00	--	--	0.00

Klebsiella pneumoniae was most sensitive to Imipenem and Piperacillin / Tazobactam (100%) followed by Ciprofloxacin, Levofloxacin, Norfloxacin and Ofloxcine (75%), Nalidixic acid, Nitrofurantion and Ceftriaxone (62.50%), Cefixime (50%) and Cephradine (25%).

Proteus mirabilis was most sensitive to Imipenem, Piperacillin / Tazobactam and Ceftriaxone (100%) whereas Cioprofloxacin, Levofloxacin, Nitrofurantion and Cefixime (50%).

Staphylococcus aureus was most sensitive to Nitrofurantion and Vancomycin (100%) followed by Piperacillin / Tazobactam and Imipenem (90%), Cephradine and Ceftriaxone (50%), Norfloxacin (33.33%), Ciprofloxacin, Levofloxacin and Ofloxacin (30%), Nalidixic acid (22%) and Cefixime (10%) (Table-II).

## DISCUSSION

The current study was carried out to find out the frequency of UTI and antimicrobial susceptibility pattern among patients with diabetes from Karachi, Pakistan. The frequency of UTI in patients with diabetes 52.76% in our study, whereas it was 34.5%, 13.8% in Nepal and Ethiopian study, respectively.^7^,^10^ This variation might be due to the geographical differences, personal hygiene practices and health education.

Majority of studies concluded the predominance of female UTI as compare to male. UTI is known as disease of female.^2^ UTI in female 87.85% in present study which is in agreement with other studies. The main reason might be anatomical predisposition as compared to male, which allow access of bacteria to the bladder and poor personal hygiene.^10^,^15^,^16^ Most of the patients with diabetes (35.18%) with UTI age ranges 51-60 in our study. In a study from Nepal, majority of patients with diabetes UTI were found between the ages of 31-40 years.^17^ This type of variation may be due to the environmental as well as cultural differences.

*E.coli* was found to be the most common among Gram negative isolate (71.96%) in this study as well as in other studies from Ethiopia, Pakistan and Bangladesh.^3^,^14^,^15^ Previous study showed high frequency of *E.coli* 80% whereas it was 65%, 49% and 60.5% in studies from Nepal and Burma, respectively.^6^,^10^,^17^,^18^ K.pn was found 7.48% in current study whereas it was as high 25.1% and 18.7% in earlier studies.^16^,^19^ Similarly 14%,13.8%,11% of K.pn isolates were found from local, Burma, and Nepal studies, respectively.^15^,^17^,^18^ The main reason for the high prevalence of E coli and Kpn in UTIs is that E coli and K pn can bind with the glycoconjugate receptors of the epithelial cells of urinary tract and can initiate the infection. The most common Gram positive isolate was S.aur 9.35% in this study similar as 9.4% previous study.^19^ Enterococcus Spp, P.mir, Candida was found 0.93%,1.87%, 5.61% in this study and 18.4%, 12.9%, 8% from related other published studies, respectively.^17^,^18^

Piperacillin / Tazobactam showed high sensitivity towards all urinary isolates in our as well as in another local study.^6^ All the isolated bacteria were 100% sensitive to nitrofurantoin in Ethiopian and Indian studies.^7^,^16^ The reason for the variation in sensivity pattern might be nature of organism and the use of antibiotics without restrictions. Gram positive isolates were showed high sensitivity (100%) towards Vancomycin in our as well as in two Indian studies.^11^,^16^ All gram positive pathogens showed least sensitivity towards Norfloxacin in present study similar to Indian study.^16^

*E.coli* showed most higher sensitivity towards Imipenem and Piperacillin / Tazobactam (100%) in current study also supported by local studies as compared to earlier studies 75%, 68.5%, 98%.^6^,^10^,^11^-^13^ It also shown high sensitivity (100%) towards Nitrofurantoin, in an Ethiopian study similarly (93.24%) in current study as well as in studies from Nepal and India, Whereas 80.3% in another studies.^7^,^16^-^19^

*E.coli* isolates shown sensitivity towards Ciprofloxacin 38.96%, Ceftriaxone 45.93%, Norfloxacin 37.33% in this study, as well as 86.3%, 81.8%, 19.4%, 55% from previous studies, 50%, 36.4% and 28.22% in India, Ethiopia and Nepal, 90.9%, 55% and 44.35%in other studies.^7^,^11^,^12^,^14^,^16^,^17^ It showed 37.33% sensitivity towards Cefixime and 40% in Ofloxacine, in present study similar as previous study, 81% in an Indian study, while 29.84% and 88% in earlier studies.^11^,^17^

K.pn isolates most sensitive to Imipenem and Piperacillin / Tazobactamin current study as well as in local and Indianstudies.^11^,^13^ It also showed 75% sensitivity towards Ciprofloxacinin, Norfloxacin 75%,60.71%, and Ofloxacine 75%, 53.57% in current study whereas least sensitivity found in previous study 57.14%, 26.4%, Norfloxacin 33.3%, 30.18%, Ofloxacine 87% in an Indian study.^11^,^12^,^16^,^17^

K.pn isolates showed 62.50% sensitivity towards Ceftriaxone, Nalidixic acid and Nitrofurantoinin present study, whereas Ceftriaxone 48.3% and 28.57%,Nalidixic acid 46.42%and 26.4%, Nitrofurantoinin 96.2% and 42.85% in studies from India and Nepal. It showed high sensitivity towards Cefixime 87% in study from India while 50% in this study.^12^,^16^,^17^

P.mir was most sensitive to Imipenem and Piperacillin / Tazobactamin, higher sensitive towards Ceftriaxone (100%), Ciprofloxacin (80.95%) and less sensitive towards Nitrofurantoin(50%) in present study, whereas it was 57.14%, 80.95% and 66.66%, 42.85% from published studies.^14^,^17^

Staphylococcus aureus was most sensitive to Nitrofurantoin (100%), than Vancomycinin this study as well as previous study.^11^,^17^ It was 90%sensitive to Piperacillin / Tazobactam, Ceftriaxone was 50%in current and in an Indian study, while 72.22% from Nepal.^3^,^17^ The reason for the variation in sensitivity pattern might be nature of organism and the use of antibiotics without restriction.

## CONCLUSION

The frequency of UTI in diabetic patients was higher in our study. *E.coli* was the most frequent pathogen responsible for UTI in patients with diabetes. Piperacillin / Tazobactam might be the drug of choice in both Gram negative and Gram positive isolates. Imipenem and Nitrofurantoin were most sensitive in Gram negative and Gram positive isolates respectively. Monitoring the antimicrobial susceptibility pattern of isolated microorganisms provides rational use of antimicrobial agents for the treatment of UTIs, avoiding the development of antibiotic-resistant urinary pathogens.

### Author’s contribution:

**KUZ:** Concept, design, literature search, interpretation of data, preparation and approval of the final manuscript.

**AHS:** Concept, design, edited and approval of the final manuscript.

**AF:** Concept, design, edited and approval of the final manuscript.

**RS:** Interpretation of data, literature search, preparation, and approval of the final manuscript.

**AB:** Interpretation of data, preparation, and approval of the final manuscript, is responsible for integrity of research.
